# Efficacy of splint therapy for the management of temporomandibular disorders: a meta-analysis

**DOI:** 10.18632/oncotarget.13059

**Published:** 2016-11-03

**Authors:** Chao Zhang, Jun-Yi Wu, Dong-Lai Deng, Bing-Yang He, Yuan Tao, Yu-Ming Niu, Mo-Hong Deng

**Affiliations:** ^1^ Center for Evidence-Based Medicine and Clinical Research, Taihe Hospital, Hubei University of Medicine, South Renmin Road, Shiyan, China; ^2^ School of Stomatology, Hubei University of Medicine, Shiyan, China; ^3^ The State Key Laboratory Breeding Base of Basic Science of Stomatology & Key Laboratory of Oral Biomedicine, Ministry of Education, School & Hospital of Stomatology, Wuhan University, Wuhan, China; ^4^ Department of Stomatology, Taihe Hospital, Hubei University of Medicine, Shiyan, China; ^5^ The State Key Laboratory Breeding Base of Basic Science of Stomatology & Key Laboratory of Oral Biomedicine, Ministry of Education, Department of Oral and Maxillofacial Surgery, School & Hospital of Stomatology, Wuhan University, Wuhan, China

**Keywords:** temporomandibular disorders, splint, maximal mouth opening, visual analogue scales of pain, meta-analysis

## Abstract

Temporomandibular disorders (TMD) are a group of clinical problems affecting temporomandibular joint (TMJ), myofascial muscles and other related structures. Splint therapy is the most commonly used approach to treatment of TMD, but its effectiveness is remains unclear. We therefore conducted a meta-analysis to evaluate the effectiveness of splint therapy for TMD in adults. The electronic databases PubMed, EMBASE, Cochrane Library, and ClinicalTrials.gov were searched for reports published up to March 31, 2016. Thirteen eligible studies involving 538 patients were identified. The results indicated that splint therapy increased maximal mouth opening (MMO) for patients with a MMO <45mm and reduced pain intensity measured using the visual analogue scale (VAS) for patients with TMD without specific description (TMDSD). Splint therapy also reduced the frequency of painful episodes for patients with TMJ clicking. No publication bias was observed, as determined with Egger's test for all outcomes. On the basis of this evidence, we recommend the use of splints for the treatment and control of TMD in adults.

## INTRODUCTION

Temporomandibular disorders (TMD) are a group of clinical problems affecting the temporomandibular joint (TMJ), myofascial muscles and other related structures [1]. There is currently no unified standard for the classification of TMD, but research diagnostic criteria for temporomandibular disorders (RDC/TMD) are the most commonly applied criteria [2]. The main signs and symptoms involve TMJ pain and clicking, myofascial or oral masticatory muscle pain, and abnormal jaw movement [3].TMD constitute a major public health problem, as they are a main source of chronic oral facial pain, interfering with daily activities [4]. These disorders are also commonly associated with other symptoms affecting the head and neck region, including headache, ear-related symptoms, cervical spine dysfunction, and altered head and cervical posture [5, 6]. Notably, signs of TMD are detected in about 60-70% of the general population, yet only about one in four people with signs are actually aware of any symptoms [7]. The etiologies of TMD are not yet clear, but contributing factors include occlusal abnormalities, psychological stress, orthodontic treatment, microtrauma, poor health and nutrition, joint laxity and exogenous estrogen [8, 9].

The main treatment options for TMD include occlusal therapy [10], psychotherapy [11], physical therapy [12], medication [13], manual therapy [14], and surgery [15]. In practice, the occlusal splint is a removable appliance, usually fabricated of resin and most often designed to cover all of the occlusal and incisal surfaces of the teeth in the upper or lower jaw. Occlusal splint therapy is most commonly used clinical approach because of its ease of use, low cost, and broad indications. A previous meta-analysis addressed the effectiveness of splint therapy for TMD, but why it is effectiveremains unclear [16]. To further explore the clinical effectiveness of splint therapy in the management of TMD in adults, we performed the present meta-analysis to elucidate the functional properties of splint therapy by comparing the clinical effects reported in all relevant randomized controlled trials (RCTs) [17].

## RESULTS

### Literature search outcome

**Figure 1:**
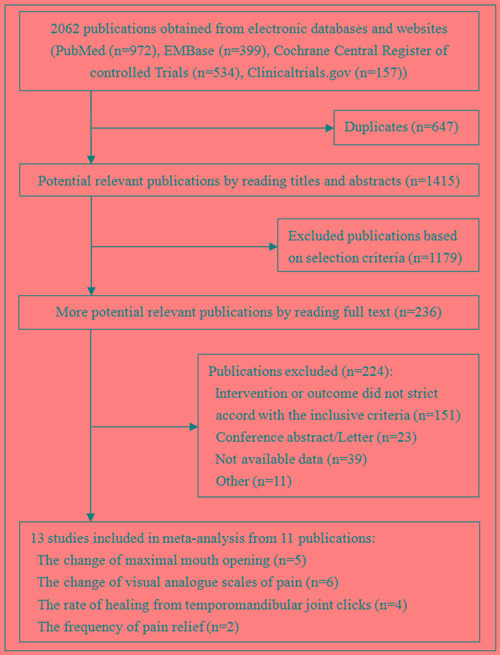
Summary of trial identification and selection

We identified 2062 publications in the electronic databases (Figure 1). Employing the selection criteria summarized in Materials and methods section, we obtained quantitative data for our meta-analysis after reading all titles, abstracts and full texts. Thirteen eligible studies [10, 18-27] from 11 publications were included in our final analysis.

**Table 1:** Search strategy and picots criteria for the systematic review

PICOS criteria	
Population	1)MeSH term: (temporomandibular joint disorders) OR (temporomandibular joint disc) OR (temporomandibular joint) OR (temporomandibular disorders) 2)Text word: (temporomandibular joint dis*) OR (dis*, temporomandibular) OR (disc*, temporomandibular joint) OR (joint dis*, temporomandibular) OR (TMJ disorders) OR (disorder, TMJ) OR (disorders, TMJ) OR (TMJ dis*) OR (temporomandibular disorder*) OR TMD
Intervention	3) MeSH term: splints OR (occlusal splints) 4) Text word: splint* OR (splints, occlusal) OR (occlusal splint*) OR (splint, occlusal)
Intervention	5) MeSH term: placebos 6) Text word: placebo* OR (no treatment) OR (sham splint*)
Outcomes	7) MeSH term:pain OR (pain measurement) 8) Text word: (maximal mouth opening) OR (MMO) OR pain OR (pain measurement) OR (visual analogue scales of pain) OR (VAS of pain) OR (healing from TMJ clicking) OR (pain relief)
Study design	9) MeSH term: randomized controlled trials AND controlled clinical trials
Search combination	1 AND 2 AND 3 AND 4 AND 5 AND 6 AND 7 AND 8 AND 9
Language	English
Electronic database	Electronic database Medline/PubMed, EMBASE, Cochrane Central Register of Controlled Trials (CENTRAL) and Clinical Trails.gov
Focused question	Is using splint therapy helpful to improve clinical outcomes in the management of temporomandibular disorders(TMD)?

Abbreviations:TMJ, temporomandibular joint; TMD, temporomandibular disorders; CENTRAL, Cochrane Central Register of Controlled Trials

### Study characteristics

The 13 eligible studies included a total of 538 patients. The change in maximal mouth opening (MMO) was determined for 122 patients from 5 studies [10, 18, 20, 23], and the initial scope of MMO was categorized to three levels: less than 37 mm, 37 mm to 45 mm, and greater than 45 mm. The change in the amount of pain experienced, as reported using the visual analogue scale (VAS), was assessed in 285 patients from 6 studies [19-22, 25]. Three types of disorder were assessed: TMD without specific description (TMDSD) [21, 22, 25], osteoarthritis [20], and TMJ clicking [19]. The rate of healing from TMJ clicking was recorded in 170 patients from 4 studies [19, 26, 27]. The frequency of pain relief was evaluated in 112 patients from 2 studies [22, 24]. There were 5 studies [18, 20, 22, 23, 25] missing standard deviations (SD), which were estimated from the P-values. Table 2 describes the clinical characteristics of patients in all 13 studies.

**Table 2:** Characteristics of the included studies

Study	Year	Region	Age (mean±SD)	Gender (female %)	Sample	Diagnostic criteria	Classification of diseases	History (month)	Intervention group	Control group	Course (month)	Follow-up (month)
Conti PC[18]	2012	Brazil	38.09/38.14	80.9%/100%	17/9	RDC/TMD	TMD	NA	splint	Self-care	NA	3
Madani AS[19]	2011	Iran	27.20±12.43/22.43±6.02	75%/92.86%	20/14	RDC/TMD	TMJ clicking	6	Splint	Physical	4/5	NA
Alpaslan C a[20]	2008	Turkey	29.8±11.1/28.9±11.3	NA	22/14	Clinical examination	Osteoarthritis	18	Hard splint	Arthrocentesis	NA	6
Alpaslan C b[20]	2008	Turkey	31.6±10.5/28.9±11.3	NA	9/14	Clinical examination	Osteoarthritis	18	Soft splint	Arthrocentesis	NA	6
Al Quran FA[21]	2006	Jardon	31.8/36	NA	38/38	Clinical examination	TMD	NA	Splint	Control appliance	NA	3
Ekberg E[22]	2003	Sweden	31/28	83.33%/90%	30/30	RDC/TMD	TMD	6	Stabilization splint	Control appliance	2.5	NA
Maloney G[23]	2002	USA	NA	NA	10/7	RDC/TMD	TMD	NA	splint	Control appliance	NA	NA
Ekberg E[24]	1998	Sweden	13-76/15-72	86.67%/96.67%	30/30	Clinical examination	TMD	36	splint	Control appliance	2.5	2.5
Wright EF[10]	1995	USA	34/31	NA	10/10	NA	TMD	NA	Soft splint	No treatment	1.5	NA
Turk DC[25]	1993	USA	35.9±9.1/33.1±8	75%/80%	30/20	NA	TMD	2	splint	BF/SM	1.5	6
Lundh H[26]	1988	Sweden	NA	NA	21/22	NA	the anterior disc displacement	NA	Flat splint	Untreated controls	6	6
Lundh H a[27]	1985	Sweden	NA	NA	24/23	NA	TMJ clicking	NA	Anterior repositioning splint	Control appliance	NA	NA
Lundh H b[27]	1985	Sweden	NA	NA	23/23	NA	TMJ clicking	NA	Flat splint	Control appliance	NA	NA

Abbreviations: RDC/TMD: research diagnostic criteria for temporomandibular disorders; TMD: temporomandibular disorders; TMJ: temporomandibular joint; BF/SM: Biofeedback-assisted relaxation and stress management; NA: Not available.

### Quality of the included studies

The risk of bias in the included studies was strictly evaluated. Details of methodological approach are presented in Table 3.

**Table 3:** Risk of bias in the included studies

Study	Year	Random sequence generation	Allocation concealment	Blinding of participants and personnel	Blinding of outcome assessment	Incomplete outcome data	Selective reporting	Other bias
Conti PC[18]	2012	unclear	high	high	high	low	low	unclear
Madani AS[19]	2011	unclear	high	high	high	low	low	high
Alpaslan C a[20]	2008	unclear	high	high	high	low	low	high
Alpaslan C b[20]	2008	unclear	high	high	high	low	low	high
Al Quran FA[21]	2006	high	high	high	high	low	low	high
Ekberg E[22]	2003	low	low	high	low	low	low	unclear
Maloney G[23]	2002	unclear	high	high	high	low	low	high
Ekberg E[24]	1998	low	low	high	low	low	low	unclear
Wright EF[10]	1995	low	high	high	high	low	low	unclear
Turk DC[25]	1993	unclear	high	high	high	low	low	high
Lundh H[26]	1988	unclear	high	high	high	low	low	high
Lundh H a[27]	1985	unclear	high	high	high	low	low	high
Lundh H b[27]	1985	unclear	high	high	high	low	low	high

### Results of individual outcome variables

#### Changes in MMO

**Figure 2:**
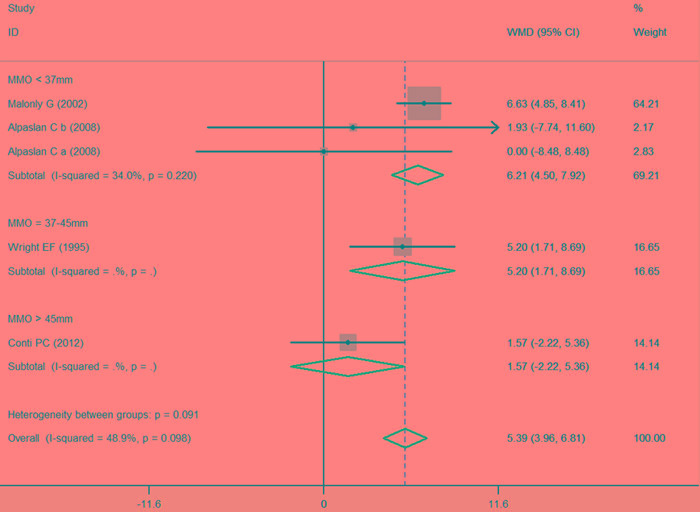
Forest plot of the changes in MMO

Comparison of the splint therapy and control groups revealed a significant difference in the change of MMO (Figure 2) (MD = 5.39, 95% CI [3.96, 6.81], I^2^ = 48.9%, *P* = 0.098]). Moreover, subgroup analysis showed that for patients with an initial MMO < 37mm (MD = 6.21, 95% CI [4.50, 7.92], I^2^ = 34.0%, *P* = 0.220) or an initial MMO = 37-45mm (MD = 5.20, 95% CI [1.71, 8.69], I^2^ = Not available (NA), *P* = NA), splint therapy led to a significant increase in MMO as compared to control. No significant difference in the change in MMO was detected for the subgroups with MMO > 45mm (MD = 1.57, 95% CI [-2.22, 5.36], I^2^ = NA, *P* = NA). In addition, for the MMO < 37mm group, meta-regression showed there was no significant difference between the control and splint therapy groups after adjusting for differences in baseline and possible confounding factors (Table 4).

**Table 4:** Meta-regression results for the main outcomes: VAS for pain and MMO <37 mm

Confounding factors	MMO <37mm			VAS of pain		
	Number of study	Coef 95%CI	*P*	Number of study	Coef 95%CI	*P*
Age	3	0.468(-1.941, 2.878)	0.703	6	-0.027 (-0.215, 0.162)	0.782
Gender	3	NA	NA	6	0.103 (-0.027, 0.234)	0.12
Region ( Ref=Europe)	0	NA	NA	1	NA	NA
North America	1	NA	NA	1	-0.300( -2.969, 2.369)	0.826
Other	2	-5.791(-12.409, 0.826)	0.086	4	0.517 (-2.146, 3.179)	0.704
Diagnostic criteria (Ref=Clinical examination)	2	NA	NA	3	NA	NA
RDC	1	5.791(-0.826, 12.409)	0.086	2	-0.193 (-1.664, 1.279)	0.797
Other	0	NA	NA	1	-0.834 (-1.853, 0.185)	0.109
Classification of diseases (Ref=Osteoarthritis)	2	NA	NA	2	NA	NA
TMD	1	5.791(-0.826, 12.409)	0.086	3	0.615 (-2.130, 3.360)	0.66
TMJ clicking	0	NA	NA	1	0.935 (-2.196, 4.066)	0.558
Course	3	NA	NA	6	-0.061 (-0.319, 0.197)	0.643
Follow-up	3	-3.217(-6.894, 0.459)	0.086	6	-0.318 (-0.659, 0.024)	0.069
Sample	3	-0.377(-0.0820, 0.067)	0.096	6	0.019 (-0.014, 0.052)	0.25
Publish year	3	-0.965(-2.068, 0.138)	0.086	6	0.053 (-0.015, 0.122)	0.131
Sources of SD(Ref=Reported SD)	1	NA	NA	2	NA	NA
Estimate the SD from *P* value	2	2.733(-10.521, 15.988)	0.686	4	-0.883 (-1.828, 0.062)	0.067

Abbreviations: VAS: Visual analogue scales, MMO: Maximal mouth opening, RDC: Research diagnostic criteria, TMD: Temporomandibular disorders, TMJ: Temporomandibular joint, SD: Standard deviation, CI: Confidence interval, Coef: Coefficient, Ref: Reference, NA: Not available.

#### Change in pain level (VAS)

**Figure 3:**
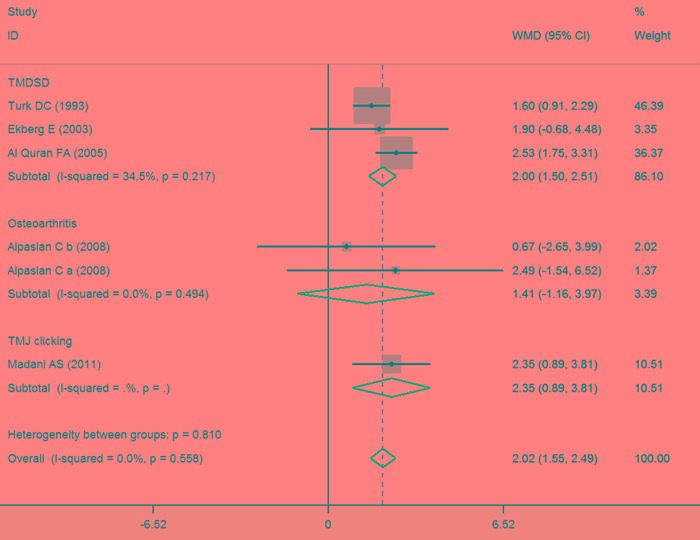
Forest plot of the changes in VAS for pain

Comparison of the splint therapy and control groups also revealed a significant difference in the changes in level of perceived pain, as determined using the VAS (Figure 3) (MD = 2.02, 95% CI [1.55, 2.49], I^2^ = 0%, *P* = 0.558). Subgroup analysis showed that patients with TMDSD in the splint therapy group experienced a significant decrease in pain as compared to control (MD = 2.00, 95% CI [1.50, 2.51], I^2^ = 34.5%, *P* = 0.217). This was also the case for patients with TMJ clicking (MD = 2.35, 95% CI [0.89, 3.81], I^2^ = NA, *P* = NA), but not for patients with osteoarthritis (MD = 1.41, 95% CI [-1.16, 3.97], I^2^ = 0%, *P* = 0.494). On the other hand, meta-regression revealed no significant differences in the change in VAS for pain after adjusting for baseline and possible confounding factors (Table 4).

#### Rate of healing from TMJ clicking

**Figure 4:**
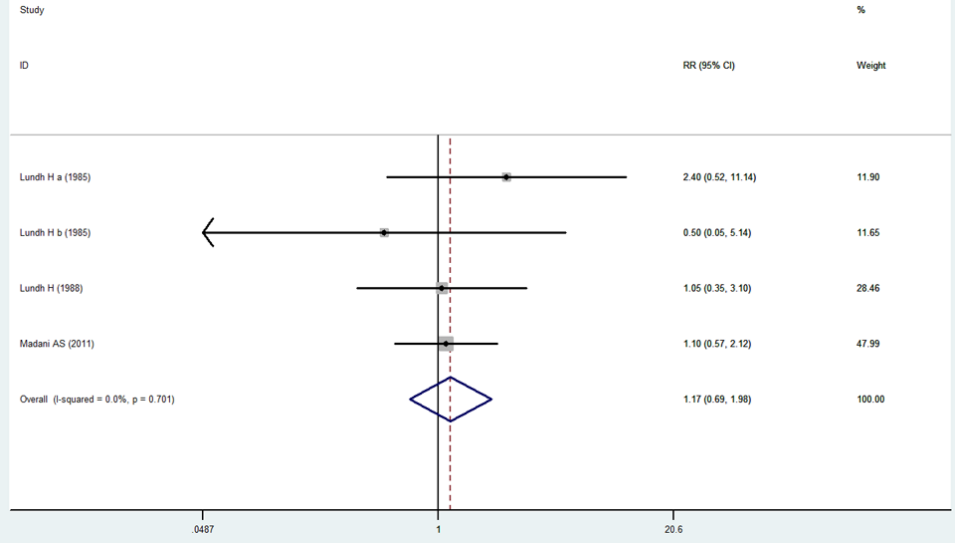
Forest plot of the rate of healing of TMJ clicking

Comparison of the splint therapy and control groups using a fixed-effects model showed that there was no significant difference in the rates of healing from TMJ clicking between the two groups (Figure 4) (RR = 1.17, 95% CI [0.69, 1.98], I^2^ = 0.0%, *P* = 0.701).

#### Frequency of pain relief

**Figure 5:**
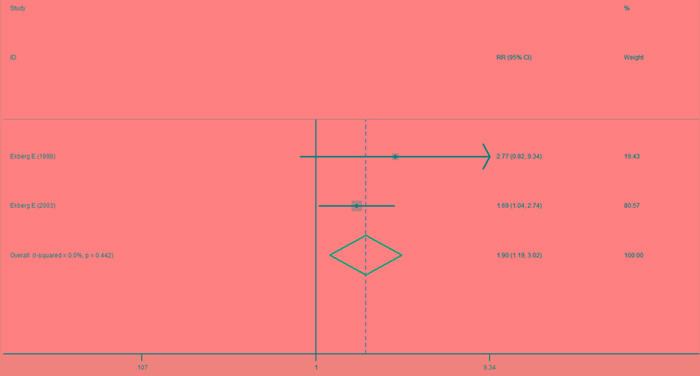
Forest plot of the frequency of pain relief

Comparison of the splint therapy and control groups using a fixed-effects model showed that splint therapy significantly reduced the frequency of painful episodes in patients with TMJ clicking (Figure 5) (RR = 1.90, 95% CI [1.19, 3.02], I^2^ = 0.0%, *P* = 0.442).

#### Publication Bias

The result of Egger's test showed there was no significant difference between both the change in MMO (Bias = -1.915 [-4.50, 0.67], *P* = 0.100) and the change in the VAS for pain (Bias = -0.072 [-2.13, 1.98], *P* = 0.927).

## DISCUSSION

In this meta-analysis, we evaluated 13 clinical trials that included TMD patients older than 18 years of age. We found that patients with an initial MMO less than 45mm showed a statistically significant change with splint therapy, whereas patients with an initial MMO greater than 45mm did not. Furthermore, subgroup analysis confirmed that splint therapy was most effective with patients exhibiting a limited ability to open their mouths. It also appears splint therapy provides significant pain relief to patients with TMDSD and TMJ clicking, but splint therapy did not reduce the pain in patients with osteoarthritis. We speculate that this is because osteoarthritis occurs after damage to the tissue has already occurred, whereas TMDSD and TMJ clicking occur in the early stage of the disease, when the splint is more able to exert a beneficial effect. In addition, although we found that splint therapy had no significant beneficial effect on the symptoms of TMJ clicking, the meta-analysis showed that splint therapy reduced the frequency of TMJ pain.

There are many types of splints for the treatment or control of TMD. These include the stabilization splint, soft splint, flat splint, and pivot splint [11]. Although the mechanisms of action of splints are not fully understood, Aleksandra et al [28] found that plasma levels of calcitonin gene related peptide (CGRP) were increased in TMD patients treated with an occlusal splint. CGRP is a potent neuropeptide thought to play an essential role in stretching and relaxing muscle, neurogenic vasodilatation and maintaining the functional integrity of peripheral tissues [29]. We therefore suggest that the increased CGRP reflects the decreased activity muscles responsible for MMO. In addition, Glaroset al [30] proposed that splints decrease pain by preventing tooth contact and reducing muscle tension. Seligman et al [31] suggested that function occlusal relationships reflect the balance of working occlusal contacts, length and symmetry of retruded contact position-intercuspal position (RCP-ICP) slides, occlusal guidance patterns, parafunction, and dental attrition. All these interacting factors play important roles during splint therapy, which explains in part why combination therapies are more effective for the treatment of TMD [32].

To our knowledge, four meta-analyses examining effectiveness of splint therapy have been published [11, 33-35]. Two of those studies [11, 34] indicated that splints reduce pain in patients with TMD. Ebrahim et al [34] included 11 studies involving 455 patients. Two outcomes, VAS for pain and the incidence of continued pain, were described without subgroup analysis. Friction et al [11] only included the rate of pain reduction among 50 patients self-reporting pain, and Al-Ani et al [35] only included pain as an outcome. MMO was not examined in any of those studies. In the present meta-analysis, we included 13 studies and used two main outcomes (VAS for pain and MMO) and two secondary outcomes (rate of healing from TMJ clicking and frequency of pain) [36]. Moreover, comprehensive subgroup analyses were conducted based on the range of initial MMO and subclasses of VAS for pain. The clinical heterogeneity was relieved, and the results were largely consistent with the experience in clinical practice [37].

There are several limitations to this study that should be addressed. First, only a few clinical trials met the inclusion and exclusion criteria. Consequently, more clinical studies will be required to confirm our results [38]. Second, some of the clinical trials had missing data on basic characteristics, possibly falsely increasing heterogeneity due to failure to perform a meta-regression for confounding factors [39, 40]. Although we estimated the missing SD from P-values, this can lead to errors [41]. Finally, although all included studies were randomized controlled trials or parallel-group design clinical trials, we could not implement complete allocation concealment, blinding the participants and personnel to the outcome assessment [42, 43].

## CONCLUSIONS

This study examined the effectiveness of splint therapy in TMD patients in a meta-analysis of published results. Our results indicate that splint therapy effectively reduces pain levels in TMDSD patients, and reduces the frequency of pain inpatients with TMJ clicking. Additionally, splint therapy increased mouth opening ability in patients with initial MMO < 45mm. On the basis of this evidence, we recommend the use of splints for the treatment and control of TMD in adults.

## MATERIALS AND METHODS

This meta-analysis was conducted according to the Preferred Reporting Items for Systematic Reviews and Meta-analyses (PRISMA) statement [44]. No ethical issues were involved in this study, and all collected data were based on published studies.

### Literature search strategy

We conducted a search of four electronic databases, PubMed, EMBASE, the Cochrane Central Register of Controlled Trials and Clinical Trails.gov, up to March 31, 2016 for eligible randomized or parallel-group design clinical trials that evaluated the effectiveness of splint therapy in patients suffering from the TMD. The electronic search and the PICO (population, intervention, comparator, outcomes) strategy are shown in Table 1.

### Selection criteria

All studies were selected in accordance with the following inclusion criteria: 1) RCTs; 2) included only TMD patients older than 18 years; 3) compared the effectiveness of splint therapy using controls receiving no treatment or placebo; 4) included only patients who should have been diagnosed with TMD (e.g., osteoarthritis, TMJ clicking or anterior disc displacement with or without reduction); 5) included patients who had not been administered a TMD treatment prior to the study; and 6) investigated one of the following outcomes: i) changes in MMO without support, ii) changes in VAS for pain, iii) rate of healing from TMJ clicking or iv) change in the frequency of pain from more than once a week to less than once a week. The main outcomes in this study were defined as a change of MMO and change in VAS for pain. The secondary outcomes were the rate of healing from TMJ clicking and the change in the frequency of pain.

### Exclusion criteria

Studies were excluded based on the following criteria: 1) pain at rest was used as the pain score; 2) the study was a duplicate; 3) the data could not be extracted or obtained through contact with the author; and 4) too little information to calculate the missing SD.

### Data extraction

The relevant information, including study design, patient characteristics, interventions, comparisons, and outcomes, were independently extracted and entered into a database by two investigators. When relevant research information was missing, particularly study design or outcome information, we contacted the original authors for clarification. The following information was extracted from each study: publication year, region, age, gender, sample, diagnostic criteria, classification of diseases, history, intervention and control groups, course, follow-up, and outcomes. Disagreements between the two investigators on data extraction or quality assessment were resolved by discussion. If the dispute persisted, other senior investigators were consulted to attain consensus.

### Quality assessment of included studies

Two investigators independently evaluated the methodological quality of eligible trials using the Cochrane collaboration tool [38] for assessing risk of bias (random sequence generation, allocation concealment, blinding of participants and personnel, blinding of outcome assessment, incomplete outcome data, selective reporting and other sources of bias).

### Statistical analysis

To describe the main outcomes based on continuous data, we used weighted mean differences (MD) [38], and 95% confidence intervals (CI). For the secondary outcomes, based on dichotomous data, we used relative risk (RR) [38, 45] and 95% CI. All the outcome data were processed using STATA 14.0 software. All missing SD were estimated from P-values [41]. We performed a statistical test for heterogeneity [37] and adopted I^2^ > 50% and P≥0.1 as evidence for heterogeneity [38]. If the data were homogeneous under a fixed-effects model, the initial scope of the MMO and disease classification were identified as key sources of heterogeneity in the main outcomes [37]. Heterogeneity was then dealt with using subgroups based on these modifiers. If the data were still heterogeneous, we introduced a random-effects model [37]. In addition, the baseline and possible confounding factors, including age, gender, region, diagnostic criteria, classification of diseases, course, follow-up, sample, publish year, and sources of SD, were detected using meta-regression [46]. Finally, the Egger's test was employed to address quantitative detection bias [47].
